# Aldo-keto Reductase Family 1 Member B 10 Mediates Liver Cancer Cell Proliferation through Sphingosine-1-Phosphate

**DOI:** 10.1038/srep22746

**Published:** 2016-03-07

**Authors:** Junfei Jin, Weijia Liao, Wenmin Yao, Rongping Zhu, Yulan Li, Songqing He

**Affiliations:** 1 Laboratory of Hepatobiliary and Pancreatic Surgery, Affiliated Hospital of Guilin Medical University, Guilin, 541001, Guangxi, People’s Republic of China; 2 Guangxi Key Laboratory of Molecular Medicine in Liver Injury and Repair, Guilin Medical University, Guilin, 541001, Guangxi, People’s Republic of China; 3 China-USA Lipids in Health and Disease Research Center, Guilin Medical University, Guilin, 541001, Guangxi, People’s Republic of China

## Abstract

AKR1B10 is involved in hepatocarcinogenesis via modulation of fatty acid and lipid synthesis. AKR1B10 inhibition results in apoptosis of tumor cells whose lipids, especially phospholipids, were decreased by over 50%, suggesting involvement of phospholipids like sphingosine-1-phosphate (S1P) in AKR1B10’s oncogenic function. Using a co-culture system, we found that co-culture of QSG-7701 (human hepatocyte) with HepG2 (hepatoma cell line) increases QSG-7701’s proliferation, in which AKR1B10-S1P signaling plays a pivotal role. Consistent with previous findings, AKR1B10 mRNA and protein levels were higher in primary hepatocellular carcinoma (PHC) tissues than in peri-tumor tissues. Interestingly, the level of S1P was also higher in PHC tissues than in peri-tumor tissues. After analyzing the correlation between AKR1B10 mRNA expression in PHC tissues and the clinical data, we found that AKR1B10 mRNA expression was associated with serum alpha-fetoprotein (AFP), tumor-node-metastasis (TNM) stage, and lymph node metastasis, but not with other clinicopathologic variables. A higher AKR1B10 mRNA expression level is related to a shorter DFS (disease free survival) and OS (overall survival), serving as an independent predictor of DFS and OS in PHC patients with surgical resection.

Primary hepatocellular carcinoma (PHC) is an extremely virulent form of tumor, causing 745,000 deaths in 2012, and is the second most common cause of cancer death in the world according to statistics gathered by GLOBOCAN^1^. Due to its late presentation, the prognosis of PHC is grim, with a median survival time of less than 2 years^2^ and a 5 year-survival rate of 15% after diagnosis (http://www.cancer.org/cancer/livercancer/detailedguide/liver-cancer-survival-rates). Thus seeking novel treatment methods and finding biomarkers for early diagnosis of PHC are beneficial to improving the survival rate of patients.

Aldo-keto reductase family 1 member B 10 (AKR1B10), also known as aldose reductase-like-1 (ARL-1), had been cloned from PHC^3^. Growing evidence has confirmed that AKR1B10 is associated with the development and progression of many cancers such as hepatocellular carcinoma^4^,^5^,^6^, smoking-related non-small-cell lung cancer^4^,^7^,^8^, esophageal adenocarcinoma^9^, gastric cancer^10^, breast cancer^11^, cervical cancer^12^, and pancreatic cancer^13^. Recently, a report implicated AKR1B10 in hepatocarcinogenes is via modulation of proliferation and apoptosis^5^. A previous study revealed that inhibition of AKR1B10 results in apoptosis of tumor cells, in which cellular lipids, especially phospholipids, were decreased by over 50%^14^. This important study prompts us to hypothesize that phospholipids are involved in AKR1B10’s oncogenic function. As a bioactive phospholipid, sphingosine-1-phosphate (S1P) is involved in cancer progression including cell transformation/oncogenesis, cell survival/apoptosis, cell migration/metastasis, and tumor microenvironment neovascularization^15^. Therefore, we hypothesized that S1P plays a pivotal role in AKR1B10’s oncogenic function. In this study, we found that co-culture of QSG-7701 (human hepatocyte) with HepG2 (hepatoma cell line) increases QSG-7701’s proliferation, and that AKR1B10-S1P signaling is necessary for this increase in proliferation; up-regulation of AKR1B10 and S1P levels was also confirmed in PHC tissues.

AKR1B10 is being considered as a biomarker candidate for PHC^10^,^16^, in that high AKR1B10 protein expression might be a favorable prognosis marker in PHC patients who have had curative hepatectomy^17^ or could reflect a less aggressive tumor behavior of PHC^16^. However, few studies have analyzed the relationship between AKR1B10 mRNA expression and PHC clinicopathological features. Here we measured AKR1B10 mRNA level by reverse transcription-polymerase chain reaction (RT-PCR) and protein expression by immunohistochemical staining or Western blotting, and then analyzed the correlation between changes in AKR1B10 mRNA expression and clinicopathologic features, or prognosis of patients with PHC.

## Results

### Co-culture with HepG2 increases QSG-7701 cell proliferation

**Figure 1:**
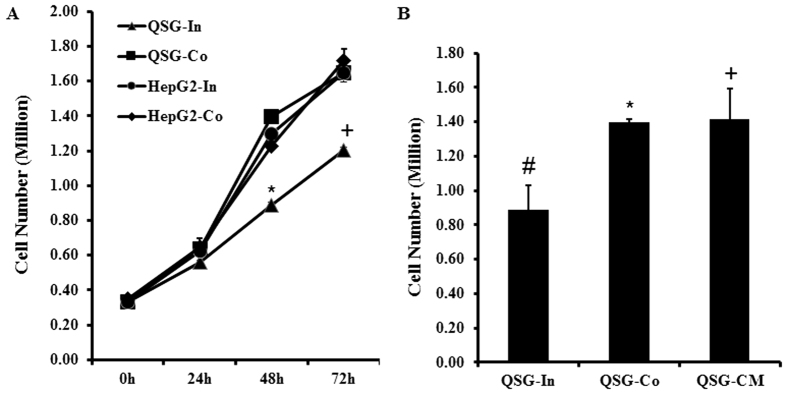
Co-culture with HepG2 increases QSG-7701 cell proliferation. (**A**) HepG2 and QSG-7701 were cultured individually (In) or co-cultured (Co), the number of viable cells of HepG2 or QSG-7701 at 24 h, 48 h and 72 h after seeding is counted by hemacytometer using 0.4% Trypan Blue Solution (QSG-In *vs* HepG2-In, or QSG-In *vs* QSG-Co, p < 0.05 at 48 h* and 72 h^**+**^). (**B**) QSG-7701 cells were cultured individually (In), co-cultured (Co) with HepG2, or cultured with the conditional medium (CM) from HepG2 for 48 h, then the number of viable cells of QSG-7701 was counted by Trypan Blue (^#^vs^*^, p < 0.05; ^#^vs^**+**^, p < 0.05; ^*^vs^**+**^, p > 0.05).

Figure 1A shows that the number of HepG2 and QSG-7701 cells increased in a time-dependent manner, but the cell number of HepG2 was higher than that of QSG-7701 at 48 h and 72 h after seeding. Thus co-culture of these two cell lines had no effect on the cell number of HepG2 but increased the proliferation of QSG-7701. Consistent with this, conditioned medium from HepG2 also increased QSG-7701 cell proliferation (Fig. 1B). Thus in subsequent experiments, in lieu of HepG2, we used conditional medium from HepG2.

### Increase in QSG-7701 cell proliferation by co-culture with HepG2 was caused by the difference in cellular AKR1B10 and not secreted AKR1B10 between two cell lines

**Figure 2:**
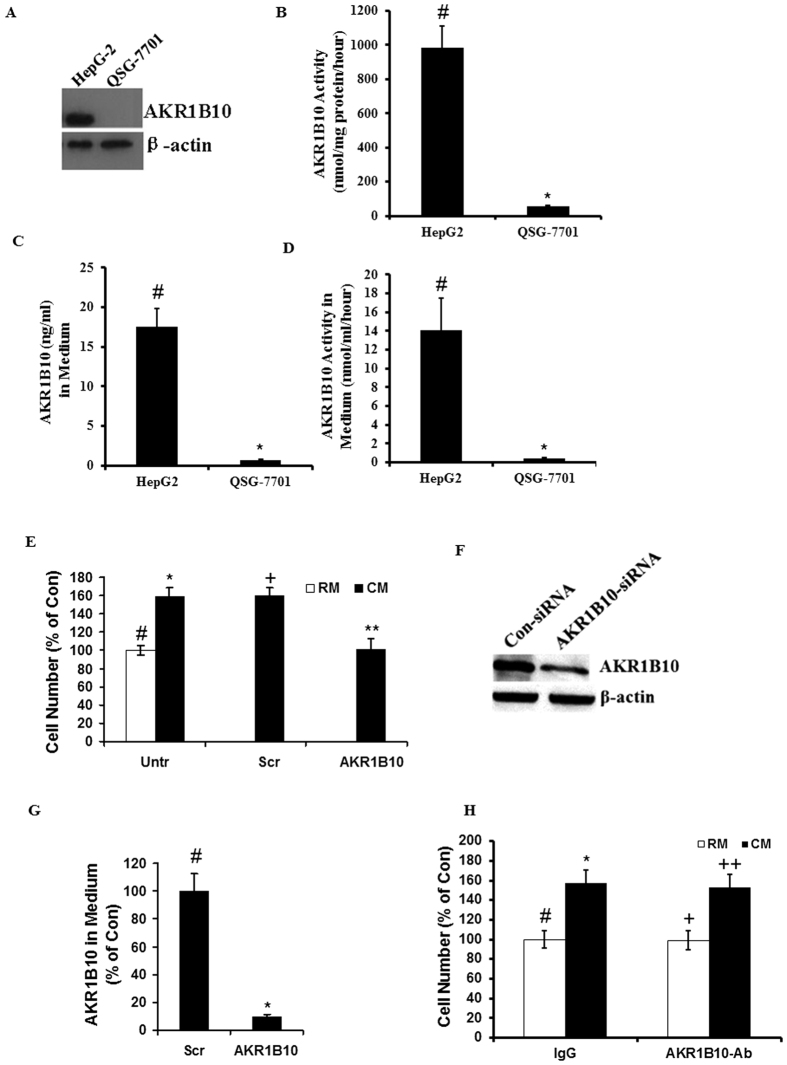
The increase in QSG-7701 cell proliferation by co-culture with HepG2 was caused by the difference in cellular and not secreted AKR1B10 between the two cell lines. (**A**) The level of cellular AKR1B10 protein in HepG2 and QSG-7701 was determined by Western Blotting. (**B**) AKR1B10 activity in cell lysate from HepG2 and QSG-7701 was investigated (^#^vs^*^, p < 0.05). (**C**) Secreted AKR1B10 in the medium from HepG2 and QSG-7701 was determined by ELISA (^#^vs^*^, p < 0.05). (**D**) AKR1B10 activity in the medium from HepG2 and QSG-7701 was investigated (^#^vs^*^, p < 0.05). (**E**) QSG-7701 cells were cultured for 48 h with the regular medium (RM) or the conditional medium (CM) from HepG2 cells transfected with scramble siRNA (Scr) or AKR1B10 siRNA (AKR1B10) or untreated (Untr) and cultured for 48 hours, then the number of QSG-7701 viable cells is counted by as above (^#^vs^*^, p < 0.05; ^#^vs^**+**^, p < 0.05; ^**+**^vs^**^, p > 0.05). (**F**) AKR1B10 inhibition by siRNA was confirmed by Western blotting. (**G**) Secreted AKR1B10 in the medium from HepG2 transfected with scramble siRNA (Scr) or AKR1B10 siRNA (AKR1B10) and cultured for 48 hours was determined by ELISA (^#^vs^*^, p < 0.05). (**H**) QSG-7701 cells were cultured for 48 h with the regular medium (RM) or the conditional medium (CM) from HepG2 cells with addition of AKR1B10 antibody or control IgG antibody (^#^vs^*^, p < 0.05; ^**+**^vs^**++**^, p < 0.05; ^*^vs^**++**^, p > 0.05).

Because AKR1B10 was reported to be involved in cell proliferation, we first investigated the difference in AKR1B10 expression between HepG2 and QSG-7701. Western Blotting revealed that AKR1B10 protein is highly expressed in HepG2 cells but non-detectable in QSG-7701 cells (Fig. 2A). Consistent with this, AKR1B10 activity was higher in HepG2 cell lysates than in QSG-7701 (Fig. 2B). Also, consistent with a previous report that AKR1B10 could be secreted^18^, we observed by ELISA that the level of AKR1B10 was higher in the medium from cultured HepG2 than that from QSG-7701 (Fig. 2C). The AKR1B10 activity was also higher in the medium from HepG2 than that from QSG-7701 (Fig. 2D). As we observed, QSG-7701 cell proliferation was increased by conditional medium from HepG2 cells or when co-cultured with HepG2. Interestingly, this enhancement could not be observed by using conditional medium from HepG2 cells whose AKR1B10 was inhibited by AKR1B10-specific siRNA (Fig. 2E). Efficiency of siRNA inhibition was confirmed by Western blotting (Fig. 2F). Although down-regulation of AKR1B10 in HepG2 cells could ameliorate AKR1B10 secretion into the medium (Fig. 2G), secreted AKR1B10 was not necessary for the increase in QSG-7701 cell proliferation by co-culture with HepG2, as this phenomenon was not blocked by AKR1B10 antibody (Fig. 2H). These results confirm that the difference in cellular and not secreted AKR1B10 between the two cell lines plays a key role in the increase in QSG-7701 cell proliferation by co-culture with HepG2.

### S1P, the downstream signaling molecule of AKR1B10, plays a key role in the increase in QSG-7701 cell proliferation when co-cultured with HepG2

**Figure 3:**
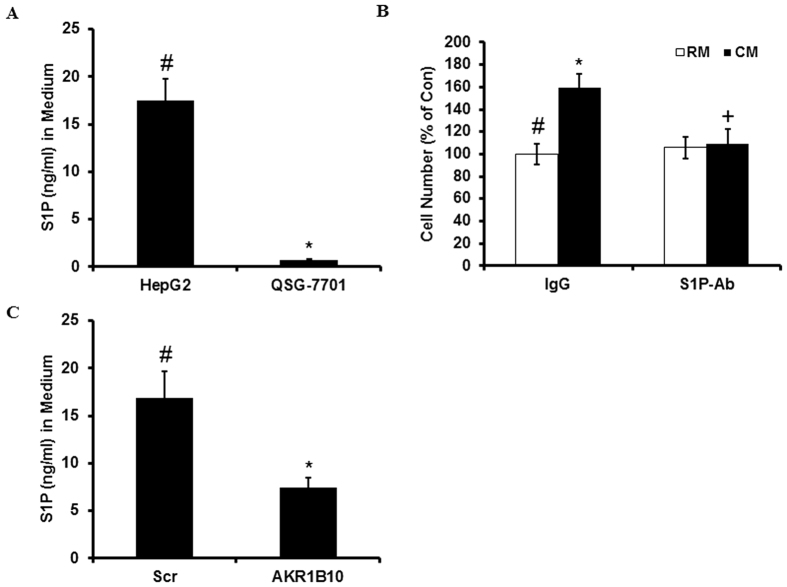
S1P is the downstream signaling molecule of AKR1B10 playing a key role in the increase in QSG-7701 cell proliferation when co-cultured with HepG2. (**A**) S1P in medium from cultured HepG2 or QSG-7701 was determined by ELISA (^#^vs^*^, p < 0.05). (**B**) QSG-7701 cells were cultured for 48 h with the regular medium (RM) or the conditional medium (CM) from HepG2 cells with addition of S1P antibody or control IgG antibody (^#^vs^*^, p < 0.05; ^*^vs^**+**^, p < 0.05). (**C**) S1P in medium from HepG2 transfected with scramble siRNA (Scr) or AKR1B10 siRNA (AKR1B10) and cultured for 48 hours was determined by ELISA (^#^vs^*^, p < 0.05).

**Figure 4:**
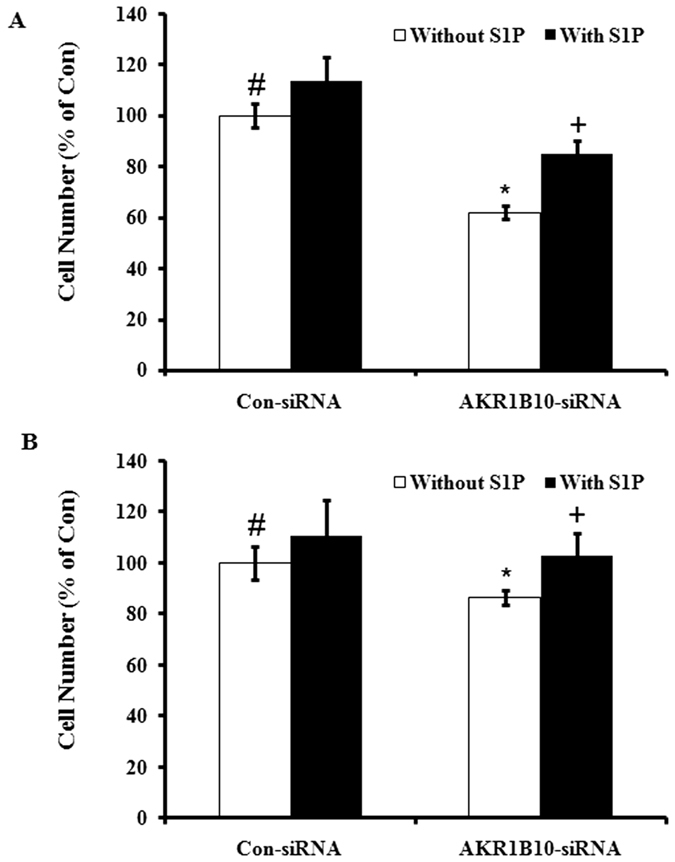
Exogenous S1P could rescue AKR1B10 downregulation-mediated cell growth inhibition in HepG2 cells. HepG2 (**A** ^#^vs^*^, p < 0.05; ^*^vs^**+**^, p < 0.05) or QSG-7701 (**B** ^#^vs^*^, p > 0.05; ^*^vs^**+**^, p > 0.05) cells were transfected with scramble siRNA (Con-siRNA) or AKR1B10 siRNA (AKR1B10-siRNA) and cultured for 24 hours, then 1 μM S1P was added and incubated for additional 48 hours. Then the number of HepG2 or QSG-7701 viable cells was counted as described above.

We observed that secreted S1P is higher in media from HepG2 cells than from QSG-7701 (Fig. 3A). In addition, increased QSG-7701 cell proliferation by conditional medium from HepG2 cells could be inhibited by S1P antibody (Fig. 3B). As expected, down-regulation of AKR1B10 in HepG2 cells could reduce S1P level in the medium (Fig. 3C). As we predicted, knockdown of AKR1B10 inhibited cell growth in HepG2 cells but not in QSG-7701, and adding S1P could reverse this inhibition in HepG2 cells (Fig. 4).

### Up-regulation of AKR1B10 and S1P in PHC

**Figure 5:**
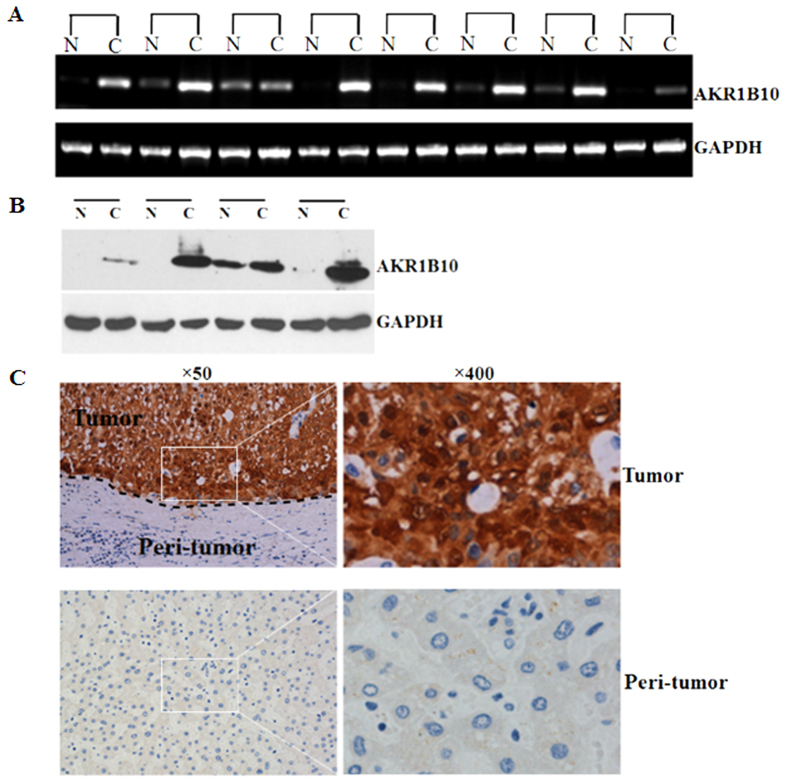
AKR1B10, S1P up-regulation in PHC tissues. (**A**) AKR1B10 and GAPDH mRNA levels were measured by RT-PCR in human PHC tissues (C) and peri-tumor tissues (N). (**B**) AKR1B10 and GAPDH protein levels were determined by Western Blotting of human PHC tissues (C) and peri-tumor tissues (N). (**C**) AKR1B10 protein level in tumor and peri-tumor tissue from a same PHC patient was investigated by IHC.

Our data suggest that signaling by AKR1B10-S1P is involved in the increase in QSG-7701 cell proliferation by co-culture with HepG2 cells. To understand whether this observation is clinically significant, we investigated AKR1B10 gene expression in PHC tissues, and observed that mRNA level of AKR1B10 was higher in human PHC tissues than in peri-tumor tissues (99 in 144, 68.8%) (Fig. 5A). Secondly, IHC results (Fig. 5C) confirmed that the protein level of AKR1B10 was higher in PHC tissues than peri-tumor tissues. Consistent with this, the positive rate of detection for AKR1B10 was higher in PHC tissues (83.0%, 39 in 47) than in peri-tumor tissues (25.5%, 12 in 47) (*p* < 0.0001). Thirdly, Western blotting confirmed the above results (Fig. 5B). In addition, using tissues from 20 patients, S1P level as measured by competitive ELISA was up to 18.51 ± 3.78 μM (mean ± SD) in PHC homogenate, which is significantly higher than that observed in peri-tumor tissues homogenate (2.92 ± 0.74 μM, *p* < 0.05).

### AKR1B10 expression level in PHC tissues and its clinical significance

**Figure 6:**
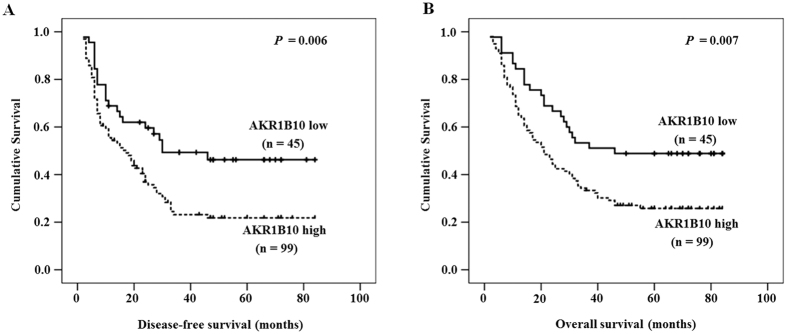
The relationship between AKR1B10 expression and DFS or OS. Patients with high AKR1B10 expression had a shorter DFS (**A**) and OS (**B**). The solid line represents the patient with low AKR1B10 expression, whereas the dashed line represents the patients with high AKR1B10 expression.

A score of 0.503 of relative AKR1B10 mRNA in cancer tissue was determined from an ROC curve obtained by MedCalc analysis based on our recent report^19^ and identified as the cut-off with the best sensitivity and the maximum specificity to predict the status of survival. After analyzing the correlation of AKR1B10 mRNA expression in PHC tissues and the clinical data (Table 1), we found that AKR1B10 mRNA expression was correlated with serum alpha-fetoprotein (AFP) level, tumor-node-metastasis (TNM) stage, and lymph node metastasis, but not with other clinicopathologic variables, such as age and gender. The data from Kaplan-Meier survival analysis revealed that a higher AKR1B10 mRNA expression level was related to a shorter DFS and OS (Fig. 6). Univariate analysis (Table 2) confirmed that the mean DFS in PHC patients with high AKR1B10 expression was 29.29 months compared with 47.04 months in PHC patients with low AKR1B10 expression, and the mean OS in the high AKR1B10 expression group was 34.80 months compared with 51.18 months in the low AKR1B10 expression cohort. In addition to AKR1B10 expression level, some clinical parameters like gender, tumor size, tumor number, TNM stage, and portal vein tumor thrombus (PVTT) were associated with DFS. The association between recurrence along with above DFS-associated clinical parameters and OS was also identified. The Cox multivariate proportional hazard model was utilized to determine whether or not some factors including AKR1B10 mRNA expression could be independent predictors of the DFS and OS of PHC patients who underwent surgical resection. Data showed that tumor size (≥5 cm), PVTT (yes) and AKR1B10 mRNA expression level (high) were independent predictors for DFS and OS (Table 3). In addition, recurrence was related to OS but not to DFS, and recurrence (yes) could be used as an independent predictor for OS (Table 3).

## Discussion

AKR1B10 is cloned from PHC^3^, and its up-regulation in PHC has been confirmed in some studies^3^,^6^. In agreement with these studies, this study showed that AKR1B10 mRNA level was higher in human PHC tissues than in peri-tumor tissues (99 in 144, 68.8%) and the protein level of AKR1B10 was also higher in PHC tissues than in peri-tumor tissues. Also, the IHC positive rate of AKR1B10 is 83.0% in PHC tissues and 25.5% in peri-tumor tissues.

Consistent with a recent report that AKR1B10 is involved in hepatocarcinogenesis by modulating cell proliferation and apoptosis^5^, we found that co-culture of QSG-7701 (human hepatocyte) with HepG2 (hepatoma cell line) increases QSG-7701’s proliferation, and this increase is modulated by a higher level of cellular AKR1B10 in HepG2 than in QSG-7701. AKR1B10 can be secreted outside^18^, but secreted AKR1B10 was not necessary for the increase in QSG-7701 cell proliferation by co-culture with HepG2 because this phenomenon was not blocked by AKR1B10 antibody. The increase in QSG-7701 cell proliferation can be observed when cultured with conditional medium from HepG2 cells, but cannot be observed if AKR1B10 in HepG2 cells was downregulated by siRNA. Thus an indirect effect by cellular AKR1B10 and not secreted AKR1B10 from HepG2 cells accounts for the increase in QSG-7701 cell proliferation by co-culture with HepG2. We speculated that S1P is the potential molecule downstream of AKR1B10 that may be directly mediating this increase in QSG-7701 cell proliferation. It is well known that secreted S1P is a bioactive phospholipid involved in cell proliferation^15^. Supporting our hypothesis, we found that secreted S1P is much higher in HepG2 than in QSG-7701, and S1P antibody could block the increase in QSG-7701 cell proliferation when cultured with conditioned medium from HepG2, and down-regulation of AKR1B10 reduced secreted S1P from HepG2. These data, together with a previous report that AKR1B10 inhibition results in apoptosis of tumor cells, in which the level of cellular phospholipids was decreased significantly^14^, prompted us to consider S1P as the potential molecule downstream of AKR1B10 that is involved in AKR1B10’s oncogenic function. It is well known that AKR1B10 knockdown inhibits proliferation of cancer cells. To better understand signaling by AKR1B10 and S1P, we added S1P to these two cell lines in which AKR1B10 was knocked down to test if exogenous S1P could rescue this inhibition of cell proliferation. As we expected, AKR1B10 knockdown inhibited proliferation of HepG2 cells but not of QSG-7701. This may be because there is more AKR1B10 protein in HepG2 than in QSG-7701, and adding S1P could rescue the inhibition in proliferation of HepG2 cells (Fig. 4). This hypothesis was also confirmed by the observation that AKR1B10 and S1P levels were much higher in PHC tissues than in peri-tumor tissues. In summary, we found that AKR1B10 is involved in cell proliferation of liver cancer through S1P. It is reported that AKR1B10 is involved in hepatocarcinogenesis via modulation of proliferation and cell apoptosis primarily through the synthesis of fatty acid, which is a substrate for sphingolipid metabolism pathway. Besides S1P, which is one of the most important signaling molecules in sphingolipid pathway, other upstream and downstream molecules of S1P and related enzymes in this pathway are also possibly implicated in this process. The association between AKR1B10 mRNA expression level and unfavorable prognosis was demonstrated in this study; however, inconsistent result was obtained in the previous studies analyzing AKR1B10 protein expression level. Therefore, 11 paired tissues from hepatocellular carcinoma were obtained to determine AKR1B10 mRNA and protein expression levels side by side (data not shown), and we found that AKR1B10 mRNA and protein expression levels are not always consistent in a same patient. The significant correlation between AKR1B10 mRNA and protein expression in hepatocellular carcinoma was not observed (*p* = 0.284), and thus, post-transcription mechanism might be involved in the role of AKR1B10 in hepatocarcenogenesis.

AKR1B10 is as a potential tumor marker^20^ in non-small-cell lung cancer^21^ and breast cancer^11^. AKR1B10 cloned from PHC has a potential to become a diagnostic and prognostic biomarker for PHC, and indeed, it was confirmed to be a marker for differentiation and proliferation of PHC^6^, an independent risk factor for PHC in chronic hepatitis C patients^22^, and a potential promising biomarker to differentiate PHCs from benign hepatic lesions^5^. Tissue micro-array data from two groups (one has 168 PHC patients and another one has 255 PHC patients) and further clinic-pathological data analysis revealed that a high AKR1B10 protein expression level reflects a less aggressive tumor behavior^16^ and thus might be a favorable prognosis marker in PHC patients who underwent curative hepatectomy^17^. Given that protein expression is not always consistent with mRNA expression, we analyzed the correlation between AKR1B10 mRNA expression level and the clinical data from 144 cases of PHC. In line with a previous result of a proportional correlation between AKR1B10 expression level and serum AFP^22^, we observed that AKR1B10 mRNA expression level associated with serum AFP. We further confirmed that the mRNA expression level of AKR1B10 correlated with TNM stage and lymph node metastasis, and that a high AKR1B10 mRNA expression level was related to a shorter DFS and OS. Thus AKR1B10 mRNA expression level is an independent predictor on DFS and OS of PHC patients who underwent surgical resection. All these data are contradictory to a recent report that high AKR1B10 protein expression level might be useful as a marker of a favorable prognosis in patients with hepatocellular carcinoma after curative hepatectomy^17^. Different results might be caused by differences in methodology or cohort of samples.

In summary, co-culture of QSG-7701 (human hepatocyte) with HepG2 (hepatoma cell line) increased QSG-7701’s proliferation, and this increase in proliferation is mediated by AKR1B10-S1P signaling. AKR1B10 and S1P expression levels were higher in PHC tissues than in peri-tumor tissues. AKR1B10 mRNA level correlated with serum AFP level, TNM stage, and lymph node metastasis. A higher AKR1B10 mRNA expression is related to shorter DFS and OS, and AKR1B10 mRNA high expression is an independent predictor in PHC patients with surgical resection. Additionally, AKR1B10-S1P is a potential therapeutic target in PHC.

## Materials and Methods

### Cells and materials

The hepatoma cell line HepG2 obtained from American Type Culture Collection (Manassas, USA) and the human hepatocyte cell line QSG-7701 purchased from the Cell Bank of Type Culture Collection of Chinese Academy of Sciences (Shanghai, China) were cultured in Dulbecco’s modified Eagle’s medium (DMEM, HyClone Corp, Logan, UT, USA) supplemented with 10% heat-inactivated fetal bovine serum (HyClone Corp, Logan, UT, USA), 100 U/ml penicillin and 100 μg/ml streptomycin in a 5% CO_2_ humidified atmosphere at 37 °C. For some experiments, HepG2 and QSG-7701 were co-cultured in Corning Transwell plates (Sigma, St. Louis, MO), in which QSG-7701 cells were grown in the lower compartment and HepG2 cells were grown in the upper compartment. These two compartments were separated by a polycarbonate membrane with 0.4 μm pore size. AKR1B10 Antibody (sc-54255) was from Santa Cruz Biotechnology, Inc (Santa Cruz, CA, USA) or from ABCAM (ab57547). AKR1B10 siRNA (sc-72342) and β-Actin Antibody (sc-130301) were also purchased from Santa Cruz Biotechnology, Inc (Santa Cruz, CA, USA). The RNeasy mini kit was bought from Qiagen (Santa Clarita, CA, USA), and the iScript cDNA synthesis kit was from Bio-Rad Laboratories, Inc (Hercules, CA, USA). S1P competitive ELISA kit was purchased from Echelon Biosciences. Lipofectamine 2000 was from Invitrogen Corp (Carlsbad, CA, USA). Other unlisted chemicals were purchased from Sigma-Aldrich Co. LLC.

### Cell growth assay

The number of viable cells was counted by hemacytometer using 0.4% Trypan Blue Solution (15250-061, Thermo Fisher Scientific Inc.) or determined by an *in vitro* toxicology assay kit (MTT-based; Sigma, St. Louis, MO, USA) as described in our previous study^23^.

### SDS-polyacrylamide gel electrophoresis and Western blotting

To obtain cell lysates, HepG2 or QSG-7701 cells were lysed in lysis buffer, and tissue samples were homogenized. These lysates were then analyzed by SDS-polyacrylamide gel electrophoresis and immunoblotting analysis as described previously^24^.

### AKR1B10 activity Assay

Cell lysate, tissue homogenate, or medium were subjected to AKR1B10 enzyme activity assays using DL-glyceraldehyde as a substrate. AKR1B10 activity was expressed as oxidized NADPH (nmol)/protein (mg)/h for cell lysate and tissue homogenate or oxidized NADPH (nmol)/medium(ml)/h for medium as described previously^18^.

### Sandwich ELISA

AKR1B10 in culture medium was quantified using Sandwich ELISA as described by Luo D *et al.*^18^.

### Blocking studies

For studies in which antibodies were used to block S1P or AKR1B10, QSG-7701 cells were treated with conditional medium from HepG2 cells in the absence or presence of S1P or AKR1B10 antibody (2.5–10 μg/ml) for 24 h. The isoform-matched control antibody was used. After the incubation, the number of viable cells was quantified as above.

### Transfection of siRNA

In order to down-regulate AKR1B10, small interfering RNA (siRNA) approach was used. The HepG2 cells were cultured for 24 h in 24-well plates before transfection with 40 nM siRNAs using Lipofectamine 2000. After 48 h incubation with AKR1B10 siRNA or Con-siRNA, the HepG2 cells and medium were collected. AKR1B10 knockdown was confirmed by Western blot analysis as above.

### S1P determination

S1P in tissue homogenate or medium was analyzed using a S1P competitive ELISA kit according to the manufacturer’s instructions.

### RT (reverse transcription)-PCR

RT-PCR reactions for AKR1B10 or GAPDH as an internal control were performed as previously described^20^. The primers used to detect AKR1B10 were 5^/^-CTGGATCCGGCAAGATTAAGGAGAT-3^/^(forward) and 5^/^-GACTGCGGCCGCGATATCCACCAGG-3^/^(reverse) and the primers for GAPDH were 5^/^-GAATTTGGCTACAGCAACAGGGTG-3^/^ (forward) and 5^/^-TCTCTTCCTCTTGTGCTCTTGCTG-3^/^(reverse).

### Immunohistochemistry (IHC)

After being blocked with 10% goat serum at room temperature for 30 min, the sections were incubated with AKR1B10 antibody at 4 °C in a moist chamber overnight, and then stained with 3,3-diaminobenzidine tetrahydrochloride (DAB) after incubation with biotinylated second antibody for 1 hour at room temperature^25^.

### The source of specimens

144 cases of tissues including PHC tissues and peri-tumor tissues were obtained from PHC patients who were diagnosed by ultrasonography, CAT scans, magnetic resonance imaging, pathological, serological, and clinical examination. These tissues were collected from Affiliated Hospital of Guilin Medical University from 2001 to 2007. The patients’ clinicopathologic parameters were included in Table 1. After collection, the tissues to be used for RT-PCR and Western blotting were frozen in liquid nitrogen immediately and then stored at −80 °C for future use. Tissues to be used for immunohistochemistry staining were fixed with 10% neutralized formalin and then embedded with paraffin. The prognosis data were collected as our previous report 25. The mean follow-up time in all selected patients after surgical resection was 36.0 months (median, 21.0 months; range, from 2.0 to 84.0 months). In this report, disease free survival was calculated from the start point (the date of surgery) to the end point (the date of recurrence, metastasis, death or last follow-up), and overall survival was calculated from the date of surgery to the date of death or last follow-up. The written informed consent was obtained from patients according to the Declaration of Helsinki, and the protocols in this study were approved by the local ethics committee.

### Statistical analysis

Data obtained from cell culture with a minimum of 3 independent experiments are expressed as the mean ± SD and analyzed by Student’s t test or ANOVA. The clinical data were analyzed using SPSS13.0 software. Correlation between AKR1B10 mRNA expression level and clinicopathologic parameters was evaluated using the Chi-square test, and quantitative variables were analyzed by the independent *t* test. The survival probability was calculated by Kaplan-Meier method, and survival curves between two groups were compared using the log-rank test. Independent predictors related to DFS were analyzed by using Stepwise Cox proportional hazard models. A value of *P* < 0.05 was considered as statistically significant.

## Additional Information

**How to cite this article**: Jin, J. *et al.* Aldo-keto Reductase Family 1 Member B 10 Mediates Liver Cancer Cell Proliferation through Sphingosine-1-Phosphate. *Sci. Rep.* **6**, 22746; doi: 10.1038/srep22746 (2016).

## Figures and Tables

**Table 1:** Correlation between AKR1B10 mRNA expression level and the clinicopathologic parameters in HCC.

Clinical character	variable	No. of patients	AKR1B10 mRNA		χ^2^	p value
			low n (%)	high n (%)		
Age (years)	<55	97	31 (32.0)	66 (68.0)	0.069	0.792
	≥55	47	14 (29.8)	33 (70.2)		
Gender	Male	122	37 (30.3)	85 (69.7)	0.316	0.574
	Female	22	8 (36.4)	14 (63.6)		
Family history	No	125	38 (30.4)	87 (69.6)	0.319	0.572
	Yes	19	7 (36.8)	12 (63.2)		
HBsAg	Negative	26	11 (42.3)	15 (57.7)	1.806	0.179
	Positive	118	34 (28.8)	84 (71.2)		
alpha-fetoprotein (ng/ml)	<100	54	23 (42.6)	31 (57.4)	5.174	**0.023**
	≥100	90	22 (24.4)	68 (75.6)		
Median size (cm)	<5	48	14 (29.2)	34 (70.8)	0.145	0.703
	≥5	96	31 (32.3)	65 (67.7)		
Cirrhosis	No	15	4 (26.7)	11 (73.3)	0.164	0.686
	Yes	129	41 (31.8)	88 (68.2)		
Tumor number	Single	90	32 (35.6)	58 (64.4)	2.071	0.150
	Multiple	54	13 (24.1)	41 (75.9)		
TNM stage	I–II	51	23 (45.1)	28 (54.9)	7.049	**0.008**
	III–IV	93	22 (23.7)	71 (76.3)		
Portal vein tumor thrombus	No	110	36 (32.7)	74 (67.3)	0.473	0.492
	Yes	34	9 (26.5)	25 (73.5)		
Lymph node metastasis	No	133	38 (28.6)	95 (71.4)	5.814	**0.016**
	Yes	11	7 (63.6)	4 (36.4)		
Recurrence	No	96	26 (27.1)	70 (72.9)	2.327	0.127
	Yes	48	19 (39.6)	29 (60.4)		

HBsAg, hepatitis B surface antigen; TNM, tumor-node-metastasis.

**Table 2:** Association between AKR1B10 expression level, clinical parameters and disease-free survival/overall survival.

Clinical character	Category	No. of patients	Disease-free survival (months)			Overall survival (months)		
			Mean	95% CI	p value	Mean	95% CI	p value
AKR1B10 expression	Low	45	47.04	36.45–57.63	**0.006**	51.18	41.51–60.84	**0.007**
	High	99	29.29	23.04–35.56		34.80	28.68–40.93	
Age (years)	<55	97	35.73	28.78–42.69	0.644	40.62	34.02–47.22	0.716
	≥55	47	33.38	23.93–42.83		38.45	29.47–47.44	
Gender	Male	122	31.73	25.90–37.57	**0.015**	36.56	30.94–42.18	**0.009**
	Female	22	53.59	38.44–68.73		58.73	45.57–71.91	
Family history	No	125	33.94	27.97–39.91	0.357	38.74	33.03–44.44	0.290
	Yes	19	40.46	25.23–55.68		47.81	33.35–62.26	
HBsAg	Negative	26	32.77	20.25–45.28	0.905	40.87	29.31–52.44	0.857
	Positive	118	35.39	29.13–41.65		39.77	33.79–45.75	
alpha-fetoprotein (ng/ml)	<100	54	34.83	25.85–43.82	0.824	41.29	32.49–50.09	0.696
	≥100	90	34.60	27.61–41.60		39.15	32.46–45.84	
Median size (cm)	<5	48	51.74	43.27–60.21	**<0.001**	60.91	52.50–69.32	**<0.001**
	≥5	96	24.52	18.71–30.32		29.50	23.75–35.25	
Cirrhosis	No	15	22.33	10.09–34.57	0.198	31.53	18.46–44.61	0.347
	Yes	129	36.22	30.23–42.21		40.86	35.15–46.58	
Tumor number	Single	90	40.98	33.57–48.40	**0.008**	45.74	38.87–52.62	**0.004**
	Multiple	54	25.14	17.51–32.77		30.33	22.57–38.10	
TNM stage	I–II	51	46.74	38.77–54.72	**<0.001**	57.59	48.92–66.27	**<0.001**
	III–IV	93	25.22	19.24–31.20		30.29	24.41–36.17	
Portal vein tumor thrombus	No	110	39.64	32.96–46.31	**0.001**	44.98	38.76–51.20	**<0.001**
	Yes	34	20.50	12.17–28.83		23.59	15.57–31.61	
Lymph node metastasis	No	133	35.08	29.21–40.95	0.916	39.92	34.32–45.52	0.961
	Yes	11	33.09	15.08–51.11		39.64	23.60–55.67	
Recurrence	No	96				32.52	26.29–38.75	**<0.001**
	Yes	48				54.82	46.22–63.43	

**Table 3:** Cox multivariate proportional hazard model of independent predictors on disease-free and overall survival.

Variable	Hazard ratio (95% CI)	*P* value
Disease-free survival		
Gender (male *vs* female)	1.426 (0.809–2.513)	0.220
Tumor size, cm (≥5 *vs* <5)	3.085 (1.690–5.630)	**<0.001**
Tumor number (multiple *vs* single)	1.048 (0.666–1.649)	0.840
TNM stage (III–IV *vs* I–II)	1.231 (0.760–1.993)	0.399
PVTT (yes *vs* no)	2.125 (1.071–4.021)	**0.032**
AKR1B10 expression (high *vs* low)	1.965 (1.187–3.256)	**0.009**
Overall survival		
Gender (male *vs* female)	1.383 (0.784–2.440)	0.263
Tumor size, cm (≥5 *vs*<5)	2.727 (1.478–5.034)	**0.001**
Tumor number (multiple *vs* single)	0.987 (0.629–1.549)	0.954
TNM stage (III–IV *vs* I–II)	1.321 (0.815–2.141)	0.259
PVTT (yes *vs* no)	2.015 (1.058–3.985)	**0.035**
Recurrence (yes *vs* no)	1.776 (1.105–2.853)	**0.018**
AKR1B10 expression (high *vs* low)	1.866 (1.127–3.091)	**0.015**

CI, confidence interval; TNM, tumor-node-metastasis; PVTT, portal vein tumor thrombus.
